# Diagnosis and Treatment of War Neuroses

**Published:** 1918-08

**Authors:** 


**Observations on the Diagnosis and Treatment of War Psycho-Neuroses Especially with Reference to Cases of Convulsions and Asthenia.** Professor LAIGNEL-LAVASTINE, Chief of the psycho-neurological Centre of the Paris Military Government, first discussed his general method of diagnosis.

1. Sufferers from war psychoneuroses are exceedingly complex patients, and it is therefore misleading to label them all with one name. Under these circumstances he has formed the habit of seeking systematically for four classes of symptoms :

(1) Physical signs of organic nervous disease;

(2) Physical signs of organic diseases, foreign to the nervous system but liable to react on it;

(3) Signs of psychical diseases;

(4) Illegitimate psychical manifestations.

The class of illegitimate manifestations includes : *allegation, utilization, perseveration, exaggeration, simulation, reclamation.*

The problem consists in the study of the innermost mental recesses of men broken in the war (their fear of returning to the front, their claims against the government in whose service they have fallen ill or been wounded, etc.). The subject must be caught redhanded in insincerity, for *de intentione homo non judicet.*

These four groups of disturbances, nervous, intrinsic, or referred, and psychic, legitimate or otherwise, are exclusively clinical in character and not founded on a pathological basis. Such grouping enables a clinical analysis of the patient to be made as simply as an analysis of a salt.

The next thing is the interpretation of each group, and the relation of each towards the others; thus the symptoms of the third group may easily result from the organic disease present in the first or second group.

2. *Application to cases of convulsions and asthenia.*

a) *Convulsions.* If one eliminates epilepsy and the hysterical crises of real insanity, the following are the principal types of convulsive phenomena :

(1) Attacks of strong emotion followed by periods of more or less dramatic anxiety.

(2) Attacks of hysteria resulting from the bursting of shells at close quarters and subsequent mental confusion; or from fainting fits arising from acute supra-renal insufficiency in conjunction with anti-typhoid vaccination.

(3) The hysterical attacks common to mental debility; and the attacks of toxic hysteria to which alcoholics are subject, with all the intermediate phases up to convulsive phases of drunkenness.

(4) The disingenuous persistence of originally legitimate hysteria, such as the anecdotal type of attack at the railway station at the time of departure of soldiers who have been on furlough and are going to rejoin their regiments at the front.

(5) The relapse, or rather resumption, on arrival, as soon as the subject has escaped neuro-psychiatric inspection.

(6) Finally the simulated attack.

If one applies to each individual case the general diagnostic scale, it will generally be easy to establish the evolutionary curve of the attacks and to place them pathogenically. It will thus be seen how some of them, by beginning physiologically, become secondarily psychogenetic. This is a course run very frequently by psychoneurotic patients. Their primary manifestations are nearly always the result of some organic trouble; they persist, become habitual, and then more and more stereotyped, though the original trouble may have completely disappeared.

b) *Asthenia.* The asthenic cases, whether predominantly psychic, nervous, or muscular, whether they appear to be an exaggerated constitutional condition or are consequent upon war injuries or some definite toxic infection, illustrate very well by the diversity of their pathogenesis the usefulness of our diagnostic scale, and at the same time lead one to the conclusion that nearly all the asthenic phenomena are organic or physiological in origin.

It is nevertheless certain that some are of psychic origin; worry, association with death, misfortune, and trouble generally, may bring about a state of asthenia by the strain on the nervous system. In these cases, however, the asthenia is the result of modifications of the internal secretions and body juices, as has been demonstrated so skilfully by certain writers among whom Major Crile is prominent.

To asthenic cases, therefore, besides the current methods, anatomical and humoral, all the physiological methods of investigation (such as sphygmomanometry, digital plethysmography, vaso-motor inscriptions, measurement of the psychomotor reactions, tests for the galvano-psychic and oculo-cardiac reflexes, etc.), must be applied in order to be certain that some physiopathological factor is not allowed to pass in cases of flagrant untruth according to psychoscopic methods, *for insincerity in certain directions does not prove that there may not be disease in others.*

3. *Therapeutic Deductions.*

The idea of almost constant association of a physiopathological and insincere element in psycho-neurotics should dominate treatment.

If the preceding considerations are borne in mind the inefficient treatment which used to be so frequent will be guarded against: i.e. *too much physical and mechanical without psychical treatment*; tardy arrival at the neurological centers; the insufficient isolation of these centers and of the patients in them; hesitation and lack of firmness in applying treatment; interference of incompetent persons; insufficient discipline; too early dismissal on convalescence or for temporary discharge from the army; faulty coordination among the different medical services so that malingerers succeed in escaping from the examiner's net.

Psycho-neurotics must therefore be treated as follows:

As soon as possible and, as far as practicable, within the army zone;

By the application of psychic methods in all cases, assisted by various other therapeutic means utilizing mechanical, physical, chemical, and biological agents;

In an *isolation hospital*, far from towns, newspapers, family, and intrigues, where a strictly therapeutic isolation, similar to that practised in the sanatoria, can be maintained, and where the moral atmosphere necessary for cure and for facilitating a state of acquiescence, re-education, and training, will depend solely upon the physician, who will not have to struggle against obstacles placed in his path by a blundering military authority and where idleness, mother of sloth, of perseveration, and of complaints can be *the most effectively* overcome by active employment of a *kind* best calculated to induce moral and physical improvement in the re-educative workshops and agricultural schools near the neurologic center.

Once these primary conditions have been fulfilled, a firm, continuous and methodical medical authority must be added (the man undertaking this work must be capable of causing his moral authority to stand clearly out over a very evident force of military discipline and a well established personal prestige). *When psycho-neurotic patients feel they are kept well in hand, they watch over their mental control.*

It is not sufficient, however, to cure the psycho-neurotics or at least to cause the disappearance of their abnormal manifestations; it is also necessary to prevent relapses as soon as the patients escape from the medical leading-strings in which they have been treated. It is for this reason that, following Henri Claude, since April 1915, he has used form letters which are sent separately to the military doctor on the return of psycho-neurotic patients to their corps.

*Conclusions.* The frequent association of organic and psychic phenomena in psycho-neurotic patients should lead to :

*A systematic attempt to diagnose one of the four classes of trouble* (nervous, intrinsic or referred, and psychic,) and *a consistent combination of psychic treatment with other therapeutics.*

For all our sick and wounded are victims of the war, and with them as with those who have suffered from factory accidents *the psychological factor is always present*; and this perpetual psychological factor is still more important with the soldier than with the factory-hand, for it does not arise merely from cupidity and idleness but, above all, *from the instinct of self-preservation.*

